# Isolation and characterization of Human Mesenchymal Stromal Cells Derived from Placental Decidua Basalis; Umbilical cord Wharton’s Jelly and Amniotic Membrane

**DOI:** 10.12669/pjms.305.4537

**Published:** 2014

**Authors:** Anahita Shaer, Negar Azarpira, Mahdokht H Aghdaie, Elaheh Esfandiari

**Affiliations:** 1 Anahita Shaer,Islamic Azad University, Fars Science & Research Branch, Fars, Iran. Transplant research Center, Shiraz University of Medical Science, Shiraz, Iran.; 2 Negar Azarpira, Transplant research Center, Shiraz University of Medical Science, Shiraz, Iran.; 3 Mahdokht H Aghdaie, Transplant research Center, Shiraz University of Medical Science, Shiraz, Iran.; 4 Elaheh Esfandiari, Transplant research Center, Shiraz University of Medical Science, Shiraz, Iran.

**Keywords:** Amnion, Mesenchymal stromal cells, Placenta, Umbilical cord

## Abstract

***Objective:*** Mesenchymal stromal cells (MSCs) are considered as an excellent source in regenerative medicine, but availability and ethical problems limited their routine use. Therefore, another available source with easy procedure and exempt from ethical debate is important. The purpose of this study is to isolate and characterize the MSCs from human placenta. The stromal cells were isolated from Placental Decidua Basalis (PDB-MSC), Umbilical cord Wharton’s Jelly (WJ-MSC) and Amniotic Membrane (AM-MSC).

***Methods:*** Full term human placentas (n=4), from cesarean section delivery were collected. Small fragments from different parts were cultures as explants. The immunophenotyping, mesodermal differentiation, growth kinetics and stemness gene expression was studied.

***Results:*** The cultivated cells from three sources expressed CD44, CD105, and CD90. Gene expression of NANOG and OCT4 confirmed the undifferentiated state. The doubling-times for WJ-MSCs, PLC-MSCs and AM-MSCs, respectively, were 21±8h, 28±9h and 25±9h at passage three and 30±5h, 45±7h and 45±7h at passage tenth. The proliferative potential of WJ-MSCs tended to be higher than the other two sources.

***Conclusion:*** The fetal derives stromal cells; especially the early passages of WJ-MSCs are available supplies for large scale production of MSC for using in clinical studies or research projects.

## INTRODUCTION

Mesenchymal stromal cell (MSC) is defined by adherency to plastic tissue culture plates and capacity to differentiate into mesenchymal lineages with certain cell surface markers. MSCs are cells with high *in vitro *self renewal capacity and ability to differentiate into multiple mesoderm, ectoderm and endoderm lineages. They also possess anti-inflammatory and immuno-modulatory effects and are considered as promising tool for cell based therapeutic strategies^1^^-^^3^ such as induction of tolerogenic response in graft vs host disease GVHD^2^^,^^3^ and enhanced antitumor therapy.^4^^,^^5^

MSCs were originally isolated from bone marrow (BM), but they are isolated from other adult tissues such as adipose, peripheral and umbilical cord blood. The aspiration of bone marrow is an invasive, relatively low cell yield (0.001%-0.01%), and with increasing age, the numbers of cells is significantly decreased.^6^^,^^7^

Placenta, as a medical waste, is usually discarded without any ethical conflict.^8^ This fetal tissue is considered as a good available source of MSCs for stem cell therapy. The human placenta is composed of amnion, chorion and deciduas.^8^

Human placenta is made up both fetal and maternal tissues. After fertilization, the human blastocyst is embedded in the endometrium which is composed of trophoblast and embryoblast. The trophoblast with maternal origin differentiates into cytotrophoblast and syncytiotrophoblast. The embryoblast with fetal origin also differentiates into the hypoblast and the epiblast. The amniotic cavity develops from epiblast and three germ layers, endoderm, mesoderm, and ectoderm are developed from hypoblst.^8^

In Anker et al. showed that amniotic membrane contains mesenchymal stem cells with osteogenic and adipogenic differentiation potential.^9^ MSCs have been isolated from other parts of the placenta including the Wharton’s jelly^10^ amniotic fluid^11^, chorionic membrane^12^. Wharton’s Jelly is the primitive connective tissue of the umbilical cord trapped in the connective tissue.^10^

We performed a study to isolate MSCs residing in the human placental decidua basalis, (PDB-MSCs), Wharton’s Jelly (WJ-MSCs), amniotic membrane (AM-MSCs) and characterize in reference to their morphology, surface phenotypes, and differentiation potential in vitro in order to obtain an alternative source of MSC for BM-MSC for therapeutic clinical applications.

## METHODS

***Tissue Processing: ***Full term normal human placentas (n=4), from cesarean section delivery, were collected from obstetric department affiliated to Shiraz University of Medical Sciences. Informed consent was obtained from mothers and the study was approved by our ethical committee. Infectious pathology was excluded by the performance of HIV, HCV and HBV tests. 

Decidua basalis and the umbilical cord were dissected from placenta. The amnion was peeled from the chorion and rinsed in phosphate-buffered saline (PBS) to remove blood.

Then the tissue was minced into small fragments (1–2 mm^3^) and were transferred to 10 cm^2^ plates containing DMEM supplemented with 10% FBS + penicillin 100 U/mL, streptomycin 100 μg/mL, and incubated at 37^oC^ containing 5% CO_2_. The culture expansion of small pieces of tissue is known as the explants method. The plates were left undisturbed for 10 days to allow the migration of cells from the margins of explants. Upon reaching 70% to 80% confluence, adherent MSC was harvested *with* trypsin and single cell suspension was used for subsequent experiments.

***Immunophenotyping: ***To phenotype cell-surface antigens, fourth-passage cells were stained with fluorescein isothiocyannate (FITC) or phycoetrythrin (PE)-conjugated antibodies specific for the following human antigens CD90-FITC, CD133-PE, CD44- FITC, CD34- FITC, and CD105-FITC (BioLegend, USA). Stained cells were analysed using FACSCalibur flow cytometer (Becton Dickinson, USA). For each sample, at least 10,000 events were recorded.^1^

***Mesodermal differentiation:*** To investigate their capacity for mesodermal differentiation adipogenic and osteogenic differentiation was carried out by culturing cells with the differentiation kit and differentiation was evaluated by Oil-Red-O and Alizarin Red staining.

***Growth Kinetic Analysis and Doubling Time: ***Four thousand cells of each cell were cultured in 6-well plates, in triplicates. The media was changed twice weekly for two consecutive weeks. The cells were harvested and subjected to trypan blue dye exclusion cell count method.

To determine the doubling time, the cells (third passage) were seeded at a density of 5×10^3^ cells/cm2. Media was changed every 3 days until cells reach confluence. Cells were harvested with trypsin-EDTA. The doubling-time was calculated using the Patterson Formula and the algorithm which was available online (http://www.doubling-time.com)

*Td=Tlg2/lg(Nt/N0); Td is the doubling time (h), T is the time taken for cells to proliferate from N0 to Nt (hour), and N is the cell count.

***RT-PCR Analysis: ***Total RNA was extracted and transcribed into cDNA. The primer pairs for amplification of OCT-4 (bp=161) and NANOG (bp=111) was:

OCT-F5'-CAGTGCCCGAAACCCACAC-3'

OCT-R5'-GGAGACCCAGCAGCCTCAAA-3'

NANOG-F5'-CAGAAGGCCTCAGCACCTAC-3'

NANOG-R5'-ATTGTTCCAGGTCTGGTTGC-3'

Amplification reactions were performed as: denaturation at 95^°C^ for 10 min ; 40 cycles of denaturation 95^°C^ for 30 s, annealing 65 ^°C^ for 40 s, and elongation 72^°C^ for 5 min. The products were electrophoresis on 3% agarose gel.

***Statistical Analysis:*** All data were presented as Mean ±SD at significance level of p ≤ of 0.05. Student T-test was performed to compare the values of two means. 

## RESULTS

**Fig.1:**
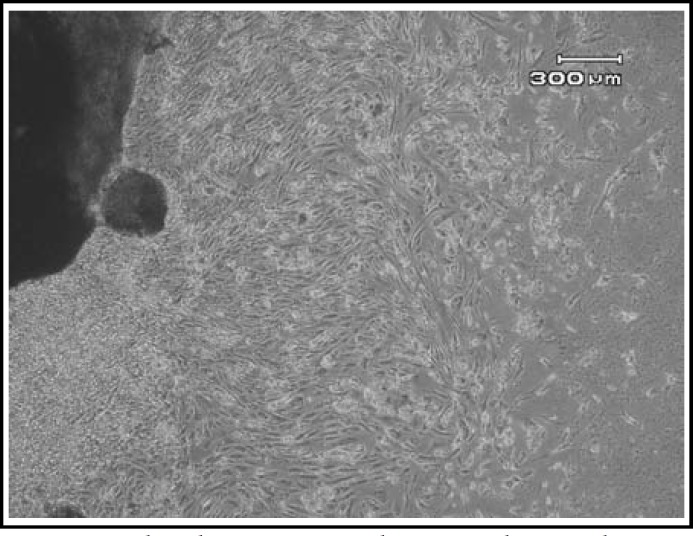
The photomicrographs were taken on day 10 - 14 of MSC in culture. The cells were grown from the edge of explants

**Fig.2:**
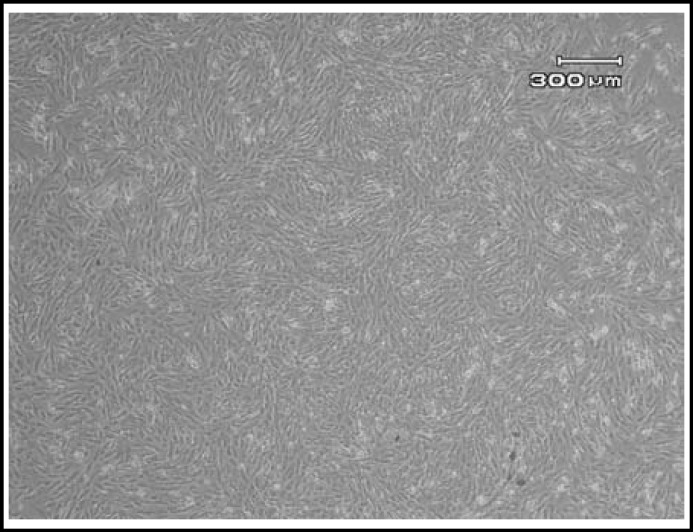
The cells reached 85-90% confluency

***Morphological features: ***The cells were immigrated from the tissue explants after 8-10 days. The initial growth of AM-MSC at primary culture (P0) consisted of adherent cells with heterogenous population; one of them was spindle shaped fibroblast-like cells and another one was epithelial-like morphology. The cells became confluent after average of 16 days. After subculture, the epithelioid cells disappeared. The WJ-MSC and PDB-MSC formed a homogenous monolayer of adherent, spindle shaped fibroblastic-like cells P0 (Fig.1). The MSCs were expanded till passage 10 (P10). Initially, the cells proliferated very rapidly with small sized spindle shaped cells morphology (Fig.2). But the features gradually changed at later passages (P9 onwards), whereby they showed morphological changes; appeared unhealthy, larger in size and eventually died. 

**Diagram.1:**
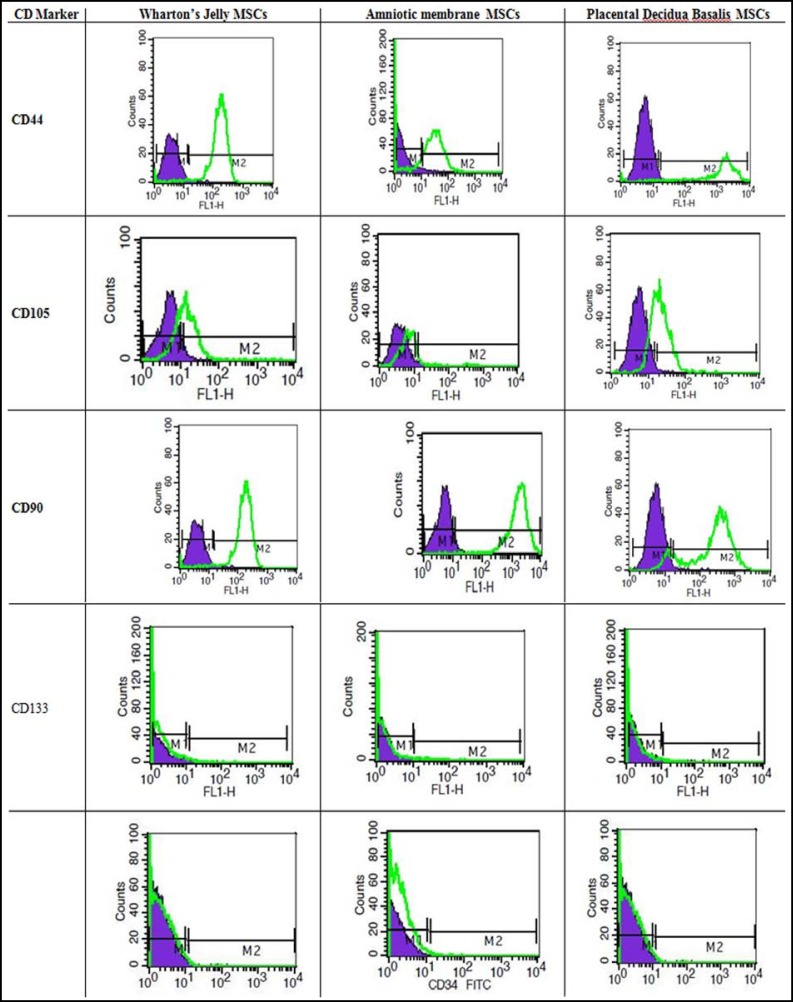
Flow cytometry histogram of MSCs from three sources for mesenchymal stem cell (CD90, CD105, CD44), hematopoietic (CD34), and endothelial (CD133) markers. Purple lines indicate background fluorescence obtained with isotype control, Green histogram: signal for each specific antibody. Results represent at least 3 experiments

***Characterization of the cells: ***The cells expressed Nanog and Oct4 transcription factors that regulate maintenance of pluripotent state. More than 95% of UC-MSC, PDB-MSC and WJ-MSC were positive for CD105, CD44 and CD90. All samples showed negative expression for CD34, and CD133 (Diagram.1). 

The cells were induced to undergo adipogenic and /or osteogenic differentiation. The accumulation of lipid vacuoles and deposition of calcium minerals was considered as adipogenic and osteogenic differentiation. The result of staining showed that all three cell types differentiated into adipogenic and osteogenic cells.

***Growth Kinetic Analysis and Doubling Time of MSC: ***Our observation suggest that doubling time of WJ -MSC falls within (20-30 hours), PDB-MSCs (30-40 hours) and AM-MSC (35-49 hours). The proliferative potential of WJ-MSCs tended to be higher than the others. The average doubling time of these cells were not significantly different. The doubling-times for WJ-MSCs, PDB-MSCs and AM-MSCs, respectively, were 21±8h, 28±9h and 25±9h at passage three and 30±5h, 45±7h and 45±7h at passage tenth. The proliferative potential of WJ-MSCs tended to be higher than that of others at these two passages.

## DISCUSSION

Our project revealed that MSC were successfully generated from human umbilical cord, amniotic membrane and deciduas basalis of placental tissues. The MSCs from these three sources were easy to obtain and also cultured at low cost. The isolated cells has similar results in both morphology and cell surface markers. The cells were not express haematopoietic (CD34, CD133) markers. All isolated MSCs were positive for endothelial progenitor (CD105), CD44 and CD90. The mesenchymal nature of isolated cells was further confirmed for their differentiation potential into mesodermal lineages after induction. After induction, the cells were successfully differentiated into adipocytes and osteocytes. The isolated cells from three sources have similar capacity for osteogenic and lipogenic differentiation. Our observations are similar to other sources of MSC.^13^

RT-PCR analysis showed expression of Nanog and Oct4 transcriptional factors which are essential in sustaining the “stemness” of a cell.^14^^,^^15^ These transcriptional factors are highly expressed in the embryonic stem cells and are essential for self renewal, pluripotency, and differentiation capacity of stem cells. ^14^ After knockdown of Sox2 in human bone marrow MSC, the multipotentiality and proliferation capacity of cells were inhibited. ^14^

Overall, the isolated MSCs from fetal origin are believed to have higher proliferative capacity than MSC from bone marrow. Fetal derived MSCs, are immunologically privileged due to expression of human leukocyte antigen G (HLA-G), MHC class I as well as low expression of MHC class II antigens. ^15^ Therefore, the MSCs can be given to any other person without rejection or need of immunosuppressive drug consumption.

In our study, the biological properties of MSCs from three sources are generally similar. However, the WJ-MSCs are obtained in larger numbers and shorter time than AM-MSCs and PDB-MSCs. Our finding was similar to Kim e al., results.^16^ They revealed that proliferative potential of WJ-MSCs tended to be higher than that of chorionic plate derived mesenchymal stem cells^16^. The MSCs from three sources were successfully cultured until passage 10-12 and beyond that, the cells underwent morphological changes, formation of vacuoles with elongated cytoplasmic process and eventually senesced. Other studies are also confirmed that in long-term, the self-renewal potency and immunosuppressive activity of MSC decreases.^17^ Vellasamy et al^8^ found that senescence of MSC cultures was evidenced by slow growth and reduced differentiation potential. 

## CONCLUSION

Overall, the fetal derives MSCs, especially the early passages of WJ-MSCs are a suitable source for large scale production of MSC in order to use in clinical studies or research projects.

## Notes

***Disclosure: ***Authors have no conflict of interests. The study was financially supported by a grant from Iran National Science Foundation (INSF).
